# High-Flow Nasal Cannula Therapy in Children With Acute Respiratory Distress With Hypoxia in A Pediatric Intensive Care UnitA Single Center Experience

**DOI:** 10.3389/fped.2021.664180

**Published:** 2021-05-07

**Authors:** Chih-Ching Chang, Yi-Chen Lin, Tzu-Chun Chen, Jainn-Jim Lin, Shao-Hsuan Hsia, Oi-Wa Chan, En-Pei Lee

**Affiliations:** ^1^Department of Respiratory Therapy, Chang Gung Children's Hospital and Chang Gung Memorial Hospital, Chang Gung University College of Medicine, Taoyuan, Taiwan; ^2^Chang Gung University School of Medicine, Taoyuan, Taiwan; ^3^Division of Pediatric Critical Care Medicine and Pediatric Neurocritical Care Center, Chang Gung Children's Hospital and Chang Gung Memorial Hospital, Chang Gung University College of Medicine, Taoyuan, Taiwan; ^4^Graduate Institute of Clinical Medical Sciences, College of Medicine, Chang Gung University, Taoyuan, Taiwan

**Keywords:** high-flow nasal cannula, child, acute respiratory distress, pediatric intensive care unit, hypoxic

## Abstract

**Aim:** High-flow nasal cannulas (HFNCs) show potential in the application of positive pressure, improving gas exchange, and decreasing work of breathing in patients with acute respiratory distress. The aims of this study were to elucidate the indications for HFNC therapy in children of all ages and diagnoses, and to evaluate the efficacy and risk factors for failure of HFNC therapy in children with acute respiratory distress with hypoxia in a pediatric intensive care unit.

**Methods:** We conducted this retrospective cohort study at a tertiary pediatric intensive care unit between January 1, 2018 and December 31, 2020. All children, from 1 month to 18 years of age, with acute respiratory distress with hypoxia and HFNC therapy were eligible. The clinical data were reviewed.

**Results:** One hundred and two children met the eligibility criteria for the study, of whom 57 (55.9%) were male, and the mean age was 7.00 6.79 years. Seventy-eight (76.5%) of the children had underlying disorders. The most common indications for the use of HFNC therapy were pneumonia (40, 39.2%), sepsis-related respiratory distress (17, 16.7%), and bronchiolitis (16, 15.7%). The failure rate was 15.7% (16 of 102 children). Higher initial and maximum fraction of inspiration O2 levels and lower initial and lowest SpO2/FiO2 (S/F) ratio were early and possible signs of failure requiring escalation of respiratory support.

**Conclusion:** In our population, we found that HFNC therapy could be initiated as the first-line therapy for various etiologies of acute respiratory distress with hypoxia in a pediatric intensive care unit and for all age groups.

## Introduction

Acute respiratory distress is the most common cause of pediatric intensive care unit admission. Invasive mechanical ventilation is an established effective supportive therapy for acute respiratory distress. However, it is associated with increased risks of nosocomial infections, lung and airway injuries, length of stay, and sedation-related complications (1,3).

High-flow nasal cannulas (HFNCs) are an increasingly used form of non-invasive respiratory support, and they have shown potential in reducing the need for intubation (4,7). HFNCs enable the administration of high concentrations of oxygen with adequate relative humidity and temperature, and they have been shown to improve airway resistance and lung compliance, achieve a certain level of continuous positive airway pressure (CPAP), eliminate dead space and decrease respiratory work (8,11). HFNC therapy has been used in infants with respiratory distress syndrome and infants with bronchiolitis, and it has been shown to decrease respiratory distress and intubation rates, increase patient comfort and ease of use compared with face masks or traditional cannulas, and shorten the length of stay in pediatric intensive care units (ICUs) (12,15).

Despite increasing evidence supporting the use of HFNCs as respiratory support for children with bronchiolitis, few studies have investigated the indications for HFNC therapy and the epidemiology of disease warranting HFNC therapy in older children in a pediatric ICU (16,25). Thus, the aims of this study were to elucidate the indications for HFNC therapy in children of all ages and diagnoses, and to evaluate the efficacy and risk factors for failure of HFNC therapy.

## Methods

This was a retrospective cohort study using chart reviews of pediatric patients who received HFNC respiratory support at the pediatric ICU of Chang Gung Children's Hospital between January 1, 2018 and December 31, 2020. Acute respiratory distress was defined as hypoxemia (SpO2 < 94%) and signs of respiratory distress despite standard-flow oxygen therapy. All patients received standard-flow oxygen therapy via a traditional nasal cannula at 15 L/min, simple mask at 610 L/min or oxygen hood with 3550% oxygen before they were switched to high flow (16, 17). The signs of respiratory distress included increased breathing rate and heart rate, color changes, grunting, nose flaring, retractions, wheezing, and sweating. The eligibility criteria for this study were: (1) age from 1 month to 18 years; and (2) patients with acute respiratory distress with hypoxia who used HFNC respiratory support for any period of time during their pediatric ICU admission. We excluded those who: (1) were older than 18 years and younger than 1 month; (2) had respiratory distress with low-flow oxygen therapy (such as a traditional nasal cannula at 15 L/min, simple mask at 610 L/min or oxygen hood with 3550% oxygen) or respiratory failure with invasive mechanical ventilation; (3) required respiratory support post extubation and after weaning from continuous positive airway pressure (CPAP) or bilevel positive airway pressure (BIPAP); and (4) had a history of long-term ventilator dependency. This study was approved by the Chang Gung Memorial Hospital Institutional Review Board (IRB number: 201801252B0C502 and 201901701B0).

### HFNC Protocol

**Figure 1:**
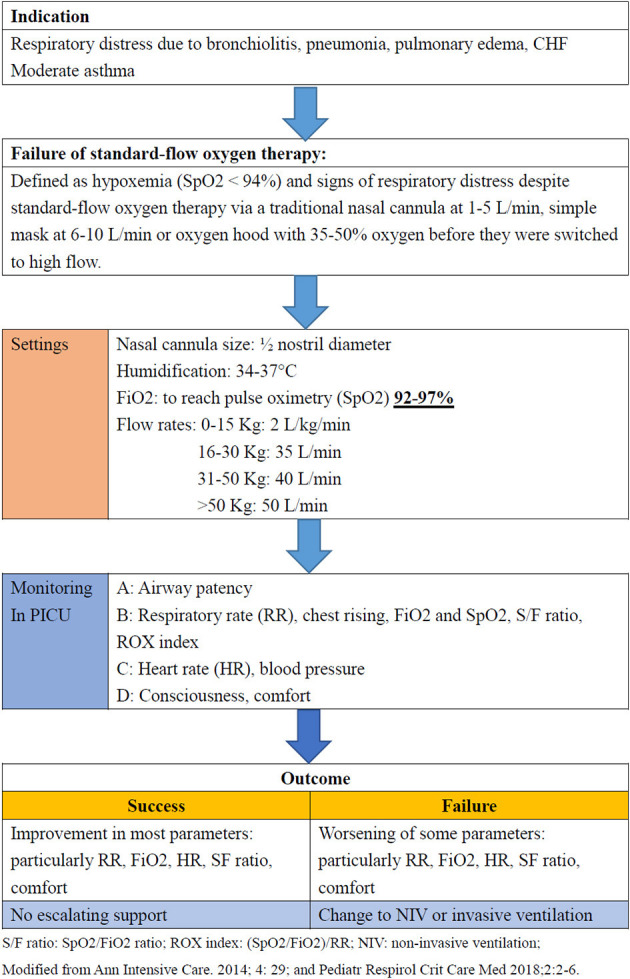
Protocol of high-flow nasal cannula therapy in the pediatric ICU at Chang Gung Children's Hospital, Taiwan.

In January 1, 2018, we initiated an institutional protocol for the use of HFNCs, which was modified from a previous study conducted in a pediatric ICU (Figure 1) (3, 6). HFNC was delivered by an Optiflow System (Fisher & Paykel, Auckland, New Zealand). The protocol includes guidelines for the indications, settings, monitoring and outcomes (success or failure) of HFNC therapy (3, 6). Fraction of inspiration O2 (FiO2) was adjusted to reach a pulse oximetry (SpO2) between 92 and 97%, and the flow setting was based on the patients' body weight: 015 kg: 2 L/kg/min; 1630 kg: 35 L/min; 3150 kg: 40 L/min; >50 kg: 50 L/min. We also monitored clinical parameters including heart rate, respiratory rate, and SpO2 as well as venous blood gas for pH and CO2. Disease severity and oxygenation were assessed according to the PRISM score, SpO2/FiO2 (S/F) ratio, and ROX index score [(SpO2/FiO2)/RR] (26, 27). The S/F ratio and ROX index score were calculated initially and every 4 h during the first 48 h after starting HFNC therapy or before stopping HFNC therapy. HFNC failure was defined as the need for escalation to non-invasive ventilation or invasive mechanical ventilation. The treating intensive care physician decided whether escalation of treatment was necessary, but it generally occurred if FiO2 > 0.6 or there was a worsening clinical state, and a similar protocol was followed in the PICU (17).

### Data Collection

The following information was collected for all patients: (1) demographics and underlying medical history; (2) primary indication and respiratory infection status; (3) clinical parameters of disease severity, including heart rate, breathing rate, SpO2, venous blood gas from a central venous catheter, including pH and PCO2, as well as Pediatric Risk of Mortality (PRISM) III score, the initial and lowest level S/F ratio and the ROX index score; (4) variables after HFNC respiratory support, including initial and maximum HFNC parameters (FiO2 and flow) and duration of HFNC use; and (5) outcomes. The primary indication was defined according to the discharge summary and treatment modalities used during the ICU stay. The primary outcome was defined as success or failure of HFNC respiratory support, and the second outcome was defined as 1-month mortality, and lengths of pediatric ICU and hospital stay.

### Statistical Analysis

The patients' characteristics including demographic and HFNC utilization data are presented as percentage (%) or mean standard deviation (SD). We divided the patients into two groups: HFNC respiratory support success, and HFNC respiratory support failure. Between-group differences were analyzed using the chi-square test or Fisher's exact test for categorical variables, and the Student's *t*-test for normally distributed continuous variables. The Mann-Whitney test was used for non-normally distributed data. Associations with outcomes between the success and failure groups were determined using univariate analysis. Receiver operating characteristic (ROC) curves for the initial and lowest S/F ratio were plotted to predict the failure of HFNC respiratory support. The respective areas under the ROC curves and cut-off values were calculated. Statistical analysis was performed using SPSS software, version 23.0 (IBM, Inc., Chicago, IL). A two-sided *p* < 0.05 was considered to be statistically significant.

## Results

### Demographics

**Figure 2:**
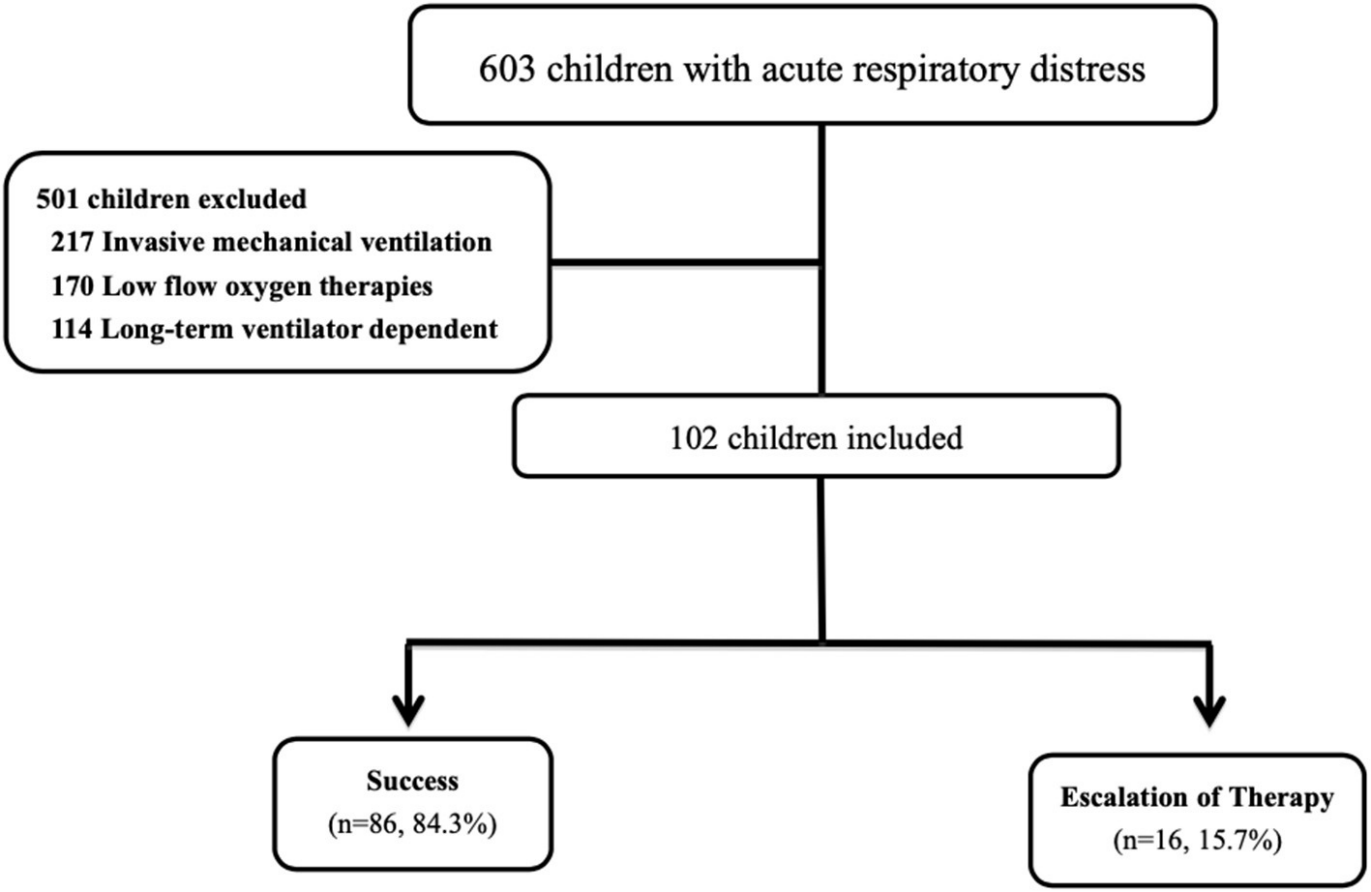
Flowchart of the included patients. During the study period, 102 children with acute respiratory distress were managed with HFNC therapy during their pediatric ICU stay. This represented 16.9% (102 of 603) of all pediatric ICU admissions due to acute respiratory distress over the same time period. The 16 (15.7%) children needed escalation of respiratory support, including five who received non-invasive ventilation and 11 who received intubation with mechanical ventilation.

During the study period, 102 children with acute respiratory distress were managed with HFNC therapy during their pediatric ICU stay (Figure 2). This represented 16.9% (102 of 603) of all pediatric ICU admissions due to acute respiratory distress over the same time period. Fifty-seven (55.9%) of the 102 children were male, and the mean age was 7.00 6.79 years. There were no significant differences in sex and age between the two groups. Seventy-eight (76.5%) of the 102 children had an underlying medical history. The most common underlying medical history was a neurologic disorder (28, 27.5%), followed by hematologic disorder/malignancy (15, 14.7%), heart disorder (13, 12.7%) and asthma/history of wheezing (7, 6.9%). The most common indication for the use of HFNC therapy was pneumonia (40, 39.2%), followed by sepsis-related acute respiratory distress (17, 16.7%) and bronchiolitis (16, 15.7%). The initial S/F ratios were 211.87 39.85 and 165.64 46.49 in the success and failure groups, respectively. After disease progression, the lowest S/F ratios were 210.07 41.72 and 147.43 49.86, respectively. There were significant differences in the initial and lowest S/F ratios between the two groups (both *p* < *0.001*). There were no other significant differences in underlying medical history, indication, PRISM III score and initial and lowest ROX index score between the two groups. The demographics of the 102 children are summarized in Table 1.

**Table 1:** Demographics of 102 children with acute respiratory distress requiring high-flow nasal cannula therapy during the study period.

**Characteristics**	**Total *N* = 102 (%)**	**Success *N* = 86 (%)**	**Failure *N* = 16 (%)**	***P*-value**
**Sex**				
Male	57 (55.9%)	45 (52.3%)	12 (75%)	0.108
Female	45 (44.1%)	41 (47.7%)	4 (25%)	
**Age group**				0.093
<23 months	28 (27.5%)	26 (30.2%)	2 (12.5%)	
24 years	24 (23.5%)	20 (23.3%)	4 (25%)	
512 years	28 (27.5%)	25 (29.1%)	3 (18.8%)	
1317 years	22 (21.6%)	15 (15.9%)	7 (43.7%)	
**Underlying medical history**				0.641
Previously healthy	24 (23.5%)	21 (24.4%)	3 (18.8%)	
Neurologic disorder (CP, epilepsy)	28 (27.5%)	24 (27.9%)	4 (25%)	
Hematologic disorder/malignancy	15 (14.7%)	10 (11.6%)	5 (31.3%)	
Asthma/history of wheezing	7 (6.9%)	6 (7.0%)	1 (6.3%)	
Cardiac disorder (pulmonary HTN, CHD)	13 (12.7%)	12 (14.0%)	1 (6.3%)	
Lung disorder (BPD, BO)	7 (6.9%)	6 (7.0%)	1 (6.3%)	
Other	8 (7.8%)	7 (8.1%)	1 (6.3%)	
**Primary indication for HFNC**				0.508
Pneumonia including aspiration	40 (39.2%)	32 (37.2%)	8 (50.0%)	
Sepsis related	17 (16.7%)	15 (17.4%)	2 (12.5%)	
Bronchiolitis	16 (15.7%)	15 (17.4%)	1 (6.3%)	
Status asthmaticus and pneumonia	5 (4.9%)	4 (4.7%)	1 (6.3%)	
Status asthmaticus	6 (5.9%)	6 (7.0%)	0 (0.0%)	
CHD with respiratory distress	9 (8.8%)	8 (9.3%)	1 (6.3%)	
Neurologic disorders, seizures	9 (8.8%)	6 (7.0%)	3 (18.8%)	
**Severity of disease**				
PRISM III score	7.76 3.49	7.53 3.40	9.36 3.90	0.104
Initial S/F ratio	205.27 73.73	211.87 39.85	165.64 46.49	<0.001^*^
Lowest S/F ratio	201.12 48.04	210.07 41.72	147.43 49.86	<0.001^*^
Initial ROX index	6.68 3.01	6.81 3.14	6.00 2.16	0.325
Lowest ROX index	6.11 2.38	6.29 2.39	5.00 2.03	0.059
**Initial HFNC parameters**				
FiO2 (%)	44.92 16.71	42.43 14.52	59.71 21.37	0.011^*^
Flow (L/min)	29.13 11.75	27.86 11.40	36.71 11.35	0.008^*^
**Maximum HFNC parameters**				
FiO2, %	46.93 18.82	43.27 15.09	68.64 24.20	0.002^*^
Flow (L/min)	30.05 12.95	28.34 11.94	40.28 14.47	0.001^*^
Flow/body weight ratio (L/kg)	1.73 0.58	1.77 0.56	1.48 0.63	0.081
**Primary outcome**				
**Escalation of therapy**	16 (15.7%)		16 (15.7%)	
Transition to non-invasive ventilation	5 (4.9%)		5 (4.9%)	
Tracheal intubation	11 (10.8%)		11 (10.8%)	
**Duration of HFNC (hours)**	65.35 75.45	71.55 78.31	24.38 30.96	0.035^*^
**Secondary outcome**				
1-month mortality	6 (5.9%)	0	6 (5.9%)	<0.001^*^
PICU LOS (days)	7.56 6.35	7.19 6.08	9.60 7.59	0.178
Hospital LOS (days)	20.08 15.90	19.44 15.69	23.86 17.22	0.339

*HFNC, high-flow nasal cannula; CP, cerebral palsy; HTN, hypertension; CHD, congenital heart disease; BPD, bronchopulmonary dysplasia; BO, bronchiolitis obliterans; PRISM, Pediatric Risk of Mortality; S/F ratio, SpO2/FiO2 ratio; FiO2, fraction of inspiration O2; PICU, pediatric intensive care unit; LOS, length of stay*.

* *Statistically significant (P < 0.05)*.

### Etiologies of Infection

Among the 102 patients, 33 had detectable pathogens (32.3%), including 13 bacterial infections from sputum cultures (7 *Haemophilus influenzae*, 4 *Streptococcus pneumoniae*, and 2 *Staphylococcus aureus*), 13 viruses [5 *Adenovirus* Ag from throat swabs or sputum specimens, 3 *Respiratory syncytial virus (RSV)* Ag from sputum specimens, 2 *Human rhinovirus/Enterovirus* PCR, 1 *Influenza A PCR*, 1 *Influenza B* PCR from throat swabs and 1 *Parainfluenza A* from a throat virus culture] and 5 *Mycoplasma pneumonia* PCR from throat swabs. In addition, two patients had combined bacterial and viral infections (*Haemophilus influenzae* and *Respiratory syncytial virus)*.

### Initial and Maximum HFNC Parameters and Clinical Parameters

After starting HFNC therapy at the pediatric ICU, the initial FiO2 and flow rates were 44.92 16.71% and 29.13 11.75 L/min, respectively. After disease progression, the maximum FiO2 and flow rates were 46.93 18.82% and 30.05 12.95 L/min, respectively. The flow/body weight ratio was 1.73 0.58 (L/kg). Table 2 summarizes the details of HFNC therapy by diagnostic indication. There were no significant differences in age, therapeutic interventions during hospitalization, and lengths of stay in the pediatric ICU and hospital between the different diagnostic indications.

**Table 2:** High-flow nasal cannula use by diagnostic indication.

**Primary indication for HFNC**	***n* (%)**	**Age (years)**	**Receiving HFNC (hours)**	**Peak FiO2 (%)**	**Peak Flow (L/min)**	**Peak Flow/kg (L/kg)**	**PICU LOS (days)**	**Hospital LOS (days)**
Pneumonia including aspiration	42 (39.2%)	8.11 7.28	73.85 98.00	43.84 15.60	31.75 11.88	1.76 0.54	7.33 5.35	19.08 13.50
Sepsis-related	17 (16.7%)	8.71 5.56	89.81 87.63	57.37 26.51	36.06 11.86	1.67 0.61	10.69 9.06	34.73 21.26
Bronchiolitis	16 (15.7%)	1.29 1.06	53.47 29.17	39.53 8.64	16.80 7.08	1.99 0.51	5.94 3.45	14.25 11.47
Status asthmaticus with pneumonia	5 (4.9%)	3.58 3.29	57.00 35.19	41.40 4.72	23.20 7.98	1.89 0.65	6.20 3.11	10.00 5.19
Status asthmaticus	6 (5.9%)	2.28 1.90	43.83 33.07	44.66 22.84	28.33 4.08	2.16 0.23	3.50 1.76	10.50 11.07
CHD with respiratory distress	9 (8.8%)	12.50 8.77	43.44 29.29	54.62 14.72	38.63 20.30	1.36 0.60	12.25 10.08	29.13 15.65
Neurologic disorders, seizures	9 (8.8%)	8.58 6.09	42.25 42.53	52.50 26.99	31.37 8.91	1.15 0.49	5.11 4.34	13.50 9.91
Total	102 (100%)	7.00 6.79	65.35 75.45	46.93 18.82	30.05 12.95	1.73 0.58	7.56 6.35	20.08 15.90

*PICU, pediatric intensive care unit; LOS, length of stay; HFNC, high-flow nasal cannula; CHD, congenital heart disease*.

The evolution of the clinical parameters and blood gas after the initiation of HFNC is shown in Table 3. There were significant improvements in heart rate, breathing rate, pulse oximetry (SpO2), S/F ratio, and ROX index score in the early HFNC period (0.58 h) and late HFNC period (824 h). No significant differences in pH and PCO2 were observed after the initiation of HFNC in the early HFNC period, however there were significant improvements in pH in the late HFNC period (824 h). No air leak syndrome or epistaxis were noted with the use of HFNCs.

**Table 3:** Evolution of clinical parameters and blood gas after initiating high-flow nasal cannula therapy.

**Parameters**	**Baseline (Before HFNC)**	**Early HFNC Period (0.58 h)[Table-fn TN3]**	***P*-value[Table-fn TN5]**	**Late HFNC period (824 h)[Table-fn TN4]**	***P*-value[Table-fn TN5]**
**Clinical parameters**					
Heart rate (beats/min)	142 (124157)	125 (110142)	<0.001^*^	128 (107144)	<0.001^*^
Breathing rate (breaths/min)	31 (2441)	28 (2433)	0.003^*^	28 (2337)	0.001^*^
SpO2 (%)	92 (8994)	99 (96100)	0.008^*^	99 (95100)	<0.001^*^
S/F ratio	230 (188235)	295.5 (244.5333.0)	<0.001^*^	291 (250333)	<0.001^*^
ROX index	6 (58)	11 (6.7513.25)	<0.001^*^	10 (713)	<0.001^*^
**Venous blood gas**					
pH	7.38 (7.347.43)	7.40 (7.367.44)	0.330	7.39 (7.337.45)	0.023^*^
PCO2 (mmHg)	40.00 (35.1047.60)	41.5 (36.4049.57)	0.133	42.75 (36.9548.57)	0.133

*Data are presented as median (IQR); IQR, interquartile range; HFNC, high-flow nasal cannula; h, hours; SpO2, pulse oximetry; S/F ratio, SpO2/FiO2 ratio*.

* *P < 0.05: statistically significant*.

*Early HFNC period data correspond to the severe values observed between 0.5 and 8 h after HFNC initiation*.

*Late HFNC period data correspond to the severe values observed between 8 and 24 h after HFNC initiation*.

*Significant difference between baseline and early HFNC period and between baseline and late HFNC period*.

### Outcomes

Most of the children (86 of 102, 84.3%) were successfully treated with HFNC during their pediatric ICU admission. The other 16 (15.7%) children needed escalation of respiratory support, including five who received non-invasive ventilation and 11 who received intubation with mechanical ventilation. The reasons for treatment failure were a rise in respiratory rate and desaturation in 13 (12.7%) children, and discontinuation of therapy due to discomfort in three (2.9%) children. Of the 16 cases who failed HFNC therapy, 11 (68.8%) failed during the first 24 h following the initiation of HFNC treatment. The mean time to failure was 24.38 30.96 h. The overall 1-month mortality rate was 5.9% (6 of 102 children), and the lengths of stay in the pediatric ICU and hospital were 7.56 6.35 and 20.08 15.90 days, respectively.

### Predictors of Failure

Among the data collected at baseline (Table 1), univariate analysis revealed that the failure group had significantly higher initial and maximum FiO2 levels than the success group (59.71 21.37 vs. 42.43 14.52%, *p* = 0.002; and 68.64 24.20 vs. 43.27 15.09%, *p* < 0.001, respectively). In addition, the initial and lowest Spo2/Fio2 ratio were also shown to be significant predictors of HFNC failure (both *p* < *0.001*). The areas under the ROCs of initial and lowest S/F ratio for HFNC failure were 0.786 and 0.816, respectively, and both cut-off S/F ratio values were 212. Therefore, higher initial and maximum FiO2 levels and lower initial and lowest S/F ratio were early and possible signs of failure requiring escalation of respiratory support. However, there were no significant differences in other baseline data, including sex, age, underlying medical history, and primary indication for HFNC.

## Discussion

In this retrospective study, we described the use of HFNC for children with acute respiratory distress at a tertiary pediatric ICU over a 3-year period. We focused on HFNC as the first-line therapy for various etiologies of acute respiratory distress with hypoxia and for all age groups. One hundred and two patients met the eligibility criteria for the study, and the failure rate was only 15.6% (16 of 102 children). In addition, there were no cases of air leak syndrome or epistaxis with HFNC therapy, Therefore, HFNC therapy appears to be a safe and effective method of non-invasive respiratory support.

### The Indications for HFNC Therapy

HFNC therapy is most commonly used for infants with acute viral bronchiolitis. However, recent studies have suggested that HFNC therapy can also be effectively and safely used in patients with a wider age range and etiologies of respiratory distress (16,25). Coletti et al. investigated the use of HFNC in 620 children with a wide range of indications in their pediatric ICU, including a significant number of subjects with status asthmaticus (41%) and congenital heart disease with respiratory distress (10%), and they reported that 10.1% of the cases needed escalation of therapy to either non-invasive ventilation or intubation with mechanical ventilation (20). In addition, Baudin et al. described 177 subjects who received HFNC therapy in a similar pediatric ICU population, including 52% with congenital heart disease, 16% with bronchiolitis, and 7% with pneumonia. They reported that HFNC therapy failure occurred in 32 cases (22%), 28 of whom required transition to non-invasive ventilation, and five required endotracheal intubation (21). Kelly et al. also reported the use of HFNC therapy in 496 children with respiratory distress in the emergency department, including 46% with bronchiolitis, 28% with pneumonia and 8% with asthma. They reported that 8% of the cases failed therapy and required intubation with mechanical ventilation following HFNC therapy (22). In our study, we also used HFNC therapy for patients with a wide range of diagnoses, including a significant number with pneumonia (39.2%), sepsis-related respiratory distress (16.7%), and acute bronchiolitis (15.7%). Of our patients, 15.7% needed escalation of therapy to either non-invasive ventilation or intubation with mechanical ventilation.

### The Risk Factors for Escalation of Therapy With the Use of HFNC Therapy

In clinical practice, it is important to have an objective method to determine if HFNC therapy is working or not. Roca et al. proposed an easy bedside tool using SaO2, FiO2 and respiratory rate to predict the success or failure of HFNC therapy, known as the ROX index (26). The authors found that a higher ROX index score was associated with HFNC success at all time points analyzed, and they concluded that a ROX index value of 4.88 at 12 h after the initiation of HFNC therapy was significantly associated with HFNC success. However, in children, predicting success using the ROX index can be much more difficult, because the respiratory rate can vary with age (27). In our study, there were no significant differences in initial and lowest ROX index scores between the two groups.

To date, few studies have assessed the risk factors for escalation of therapy to either non-invasive ventilation or intubation with mechanical ventilation, because most of the patients included in these studies have had a variety of indications and did not have severe forms of acute respiratory distress. Kelly et al. reported that failure occurred in the more critical children who presented to the pediatric emergency department with a triage respiratory rate greater than the 90th percentile for age, initial venous PCO2 >50 mm Hg and pH >7.30 (significant respiratory acidosis). A diagnosis of acute bronchiolitis seemed to be protective with respect to intubation following HFNC therapy (22). Kamit et al. reported that a lower SpO2/FiO2 (S/F) ratio at admission was a predictor of HFNC failure, and that achieving S/F > 200 at 60 min significantly predicted successful HFNC therapy (23). Betters et al. also reported that high FiO2 requirement, history of intubation, and cardiac co-morbidities were predictors of HFNC failure (24). Abboud et al. retrospectively analyzed children with viral bronchiolitis who failed HFNC (needing intubation) compared to children who were successfully treated with HFNCs, and found that improved respiratory rate and clearance of repeat pCO2 were predictors of success (25). In our study, higher initial and maximum FiO2 levels and lower initial and lowest S/F ratio were early and possible signs of failure requiring escalation of respiratory support. Therefore, these findings may help guide clinicians who would prefer to use HFNC therapy and avoid a delay in escalating therapy to either non-invasive ventilation or intubation with mechanical ventilation in children at a higher risk of failing HFNC therapy.

### Limitations

There are some limitations to this study. First, this is a retrospective study with a limited cohort of children with acute respiratory distress receiving HFNC therapy at a single center. However, very few reports in the pediatric literature have reported HFNC therapy as initial respiratory support in children with acute respiratory distress, especially for pneumonia and sepsis-related respiratory distress. Experience with HFNC therapy for this indication is particularly lacking, and this is a strength of this study. Second, because few studies have assessed the use of HFNCs and the risk of intubation in children, there is low evidence or no guidelines for the escalation of treatment to CPAP or intubation. In our study, the criteria of escalating therapy from HFNC to either non-invasive ventilation or intubation with mechanical ventilation are different in different clinical scenarios. This may have influenced the failure rate, which may limit comparisons with other studies in this field. Third, broad age groups with a small number of cases may further limit the findings of this study. Fourth, in our study, most of the severe cases (217 of 501, 43.3%) of respiratory failure were not initially treated with HFNCs, but received invasive mechanical ventilation. Only 11 patients with borderline moderate to severe respiratory failure initially received HFNC therapy, and they were finally intubated. Because HFNC therapy is being increasingly used in our hospital, the overenthusiastic use leading to delayed intubation cannot be ruled out in this study. Fifth, HFNC has been reported to fail to offer adequate PEEP, even at higher flows, for patients with moderate to severe acute respiratory distress syndrome (28). In our study, FiO2 requirement (initial or maximum) > 60% was a predictor of HFNC failure. The safety and effectiveness of providing high FiO2 (>60%) with HFNCs without adequate PEEP, given the risk of oxygen-induced lung damage at high concentrations must be considered.

## Conclusions

HFNC was used frequently over the 3-year study period for children with a wide range of ages and for a variety of indications. We found that HFNC could be initiated as the first-line therapy all age groups of children with various etiologies of acute respiratory distress in our pediatric ICU. Further prospective studies are needed to confirm the efficacy of HFNC therapy and to evaluate the risk factors of failure in different settings.

## Data Availability Statement

The raw data supporting the conclusions of this article will be made available by the authors, without undue reservation.

## Ethics Statement

This study was approved by the Chang Gung Memorial Hospital Institutional Review Board (IRB number: 201801252B0C502 and 201901701B0). Written informed consent to participate in this study was provided by the participants' legal guardian/next of kin.

## Author Contributions

C-CC and J-JL: conceptualization and writingoriginal draft preparation. S-HH, Y-CL, and O-WC: methodology. O-WC: software. J-JL and O-WC: formal analysis. Y-CL, T-CC, O-WC, and E-PL: data curation. J-JL: writingreview and editing. J-JL and S-HH: supervision. All authors contributed to the article and approved the submitted version.

## Conflict of Interest

The authors declare that the research was conducted in the absence of any commercial or financial relationships that could be construed as a potential conflict of interest.
